# Ovarian tissue cryopreservation: Low usage rates and high live‐birth rate after transplantation

**DOI:** 10.1111/aogs.13735

**Published:** 2019-10-08

**Authors:** Ellen J. Hoekman, Leoni A. Louwe, Maxime Rooijers, Lucette A. J. van der Westerlaken, Nicole F. Klijn, Gonneke S. K. Pilgram, Cornelis D. de Kroon, Carina G. J. M. Hilders

**Affiliations:** ^1^ Department of Gynecology and IVF Leiden University Medical Center Leiden The Netherlands; ^2^ Department of Gynecology Reinier de Graaf Hospital Delft The Netherlands

**Keywords:** cryopreservation, fertility preservation, ovarian tissue, premature ovarian insufficiency, ovarian tissue transplantation

## Abstract

**Introduction:**

The likelihood of survival after cancer treatment among young women with cancer has increased considerably, quality of life after treatment has drawn more attention. However, in young fertile women, fertility preservation is an important issue with regard to quality of life. One of the options of fertility preservation is ovarian tissue cryopreservation. The purpose of this follow‐up study is to present our clinical experiences and evaluate the long‐term follow up of ovarian cryopreservation to improve future patient selection.

**Material and methods:**

From July 2002 to December 2015 at the Leiden University Hospital, the Netherlands, 69 young women underwent ovarian tissue cryopreservation when they were at risk of iatrogenic premature ovarian insufficiency. Follow‐up data with regard to ovarian function were obtained until October 2018, from medical records and questionnaires.

**Results:**

Of the 69 women in whom ovarian tissue cryopreservation was performed, 12 died (15.9%), 57 were approached to participate, of which 6 were lost to follow up. The indications for ovarian tissue cryopreservation were malignant (81.1%) and benign (18.9%) diseases in which gonadotoxic treatment was scheduled. In total, twenty women (39.2%) are known to have premature ovarian insufficiency due to gonadotoxic treatment. Fifteen women conceived spontaneously, and delivered 25 babies. In this cohort, the usage rate of autotransplantation is 8.7% (7/69). In total, nine autotransplantations of cryopreserved ovarian tissue were performed in seven patients (of which 1 ovarian tissue cryopreservation was performed in another hospital) after which 6 babies were born to four women, giving a live‐birth rate of 57%.

**Conclusions:**

Ovarian tissue cryopreservation followed by autotransplantation is an effective method to restore fertility (live‐birth rate of 57%). The usage rate of 8.7% (6/69) indicates that more knowledge about the risk of premature ovarian insufficiency after gonadotoxic treatment is needed to be able to offer ovarian tissue cryopreservation more selectively.

**Key message**

Ovarian tissue cryopreservation has a low usage rate of 8.7% but a high success rate after autotransplantation of 86% restoration of ovarian function and 57% live‐birth rate.

**Abbreviations**
OTC: ovarian tissue cryopreservation
POI: premature ovarian insufficiency

## 1 INTRODUCTION

Improved cancer treatment has resulted in decreased overall cancer mortality rates. From 1991 to 2006, overall cancer death rates decreased by 12.3%. This allows. and necessitates. young women to consider quality‐of‐life issues such as fertility preservation. Especially as the Dutch cancer center has indicated that in 2017, 4.8% of all cancers were diagnosed in women under the age of 40, the need for safe and effective fertility preservation is mandatory.^1^, ^2^

It is well known that chemotherapy regimens as well as radiotherapy may compromise future fertility.^3^ The risk of premature ovarian insufficiency (POI) depends on various factors such as age, type and dose of cytotoxic therapy, and ovarian radiation dosage. As a result of the increasing emphasis on fertility issues of young survivors, ovarian tissue cryopreservation (OTC) has been developed as 1 of the options to preserve fertility in case of gonadotoxic treatment. When the patient is free of the disease and is diagnosed with POI, cryopreserved ovarian tissue can be autotransplanted to restore fertility. Over the last 2 decades, OTC has developed from the first successful report of a live birth in sheep, to the resumption of follicular activity and menstrual cycles after orthotopic autotransplantation in humans described by Oktay and Karlikaya. Since then, this technique has resulted in more than 130 healthy infants.^4^, ^5^, ^6^, ^7^

Cryopreservation of ovarian tissue is proposed as a fertility preservation option for various indications in a growing number of centers around the world. OTC benefits not only those with oncological diseases, but also girls and women with benign diseases such as β‐thalassemia and autoimmune diseases requiring bone marrow transplantation, and benign ovarian diseases that require oophorectomy (ie endometriosis) with risk of POI. Finally, OTC can be used in women with genetic disorders associated with POI.^8^, ^9^, ^10^, ^11^

Despite the large series of OTC that have been performed, the number of women in whom autotransplantation of cryopreserved ovarian tissue has been performed is low. Some research groups have described the safety and usefulness of ovarian cryopreservation to preserve fertility.^12^, ^13^, ^14^ For example, Rosendahl et al described a detailed analysis of women undergoing OTC with a 4.6% usage rate of cryopreserved ovarian tissue,^15^, ^16^ and Jadoul et al^11^ reported that 31.5% of women had POI after OTC performed before gonadotoxic treatment, with a usage rate of 3.9% and a live‐birth rate after autotransplantation of 31%. Unfortunately, the lack of international registers, (like the FertiPROTEKT network^9^) and the fact that many centers have not yet reported their results, lead to little knowledge on the precise fertility outcome of women after OTC. In this paper, we present the first study in the Netherlands, concerning the follow up and clinical experiences of women undergoing OTC and autotransplantation.

## 2 MATERIAL AND METHODS

### 2.1 Patients

From July 2002 to December 2015, 69 women and young girls underwent OTC to preserve fertility and ovarian function at Leiden University Medical Center, the Netherlands. OTC was offered to women who were at high risk of iatrogenic POI (>50%),^17^, ^18^, ^19^, ^20^, ^21^ had minimal risk of ovarian involvement of their primary malignancy,^22^ were <36 years of age, and had normal uterus and ovaries on pelvic ultrasound. Women who had been treated with gonadotoxic agents previously, who had an expected 5‐year survival rate of <50%, who were already known to have POI, in whom surgery was contraindicated and/or who were infected by human immunodeficiency virus or hepatitis B/C viruses were not offered OTC.^23^

All patients were informed about the experimental procedure of ovarian cryopreservation, with no guarantees of fertility restoration or live birth and the small risk of reintroducing malignant cells when the ovarian tissue has to be thawed and transplanted into the patient. The study was approved by the local ethics committee and the national societies of gynecology and embryologists.

### 2.2 Ovarian tissue cryopreservation

To harvest ovarian tissue, unilateral oophorectomy was performed under general anesthesia by laparoscopy or at open surgery depending on the individual situation.

In the operating room, the ovarian cortex was dissected and sliced into small pieces (10 × 5 × 1 mm), according to the description of Radford in 2001. The slices of ovarian cortex were transferred into vials and transported to the assisted reproductive technology laboratory, where a slow freezing protocol was used to cryopreserve the slices.^24^ From 2012, the whole ovary was transported to the assisted reproductive technology laboratory, where it was processed according to the Denmark technique and subsequently cryopreserved using a slow freezing protocol.^16^

### 2.3 Follow up

Follow up to determine survival, course of the disease, ovarian function, and fertility over time was obtained until October 2018 by consulting the referring doctor and by a review of medical records from the referring hospital and/or our own electronic patient dossier.

Additionally, a questionnaire was sent to all patients, addressing the following topics: current age, received gonadotoxic treatment, ovarian function, contraception method, diagnostics, hormone replacement therapy and pregnancies.

### 2.4 Ovarian transplantation technique

If a patient requested autotransplantation of ovarian tissue, the treating oncologist/hematologist was contacted for approval. The tissue was thawed as described by Rosendahl et al,^16^ followed by the operation as previously described by Andersen et al.^25^ Whenever possible the tissue was transplanted under the cortex of the remaining ovary left in situ and in some cases, into peritoneal pockets on the anterior abdominal wall and the lateral pelvic wall.

### 2.5 Statistics

POI was defined as 1 measurement of follicle‐stimulating hormone >40 IU/L (with or without the measurement of estradiol) and/or when the woman did not menstruate after treatment. Collected data were analyzed using SPSS version 25 (IBM, Armonk, NY, USA) to perform descriptive statistics and to determine the 5‐year survival rate with the Kaplan‐Meijer method.

### 2.6 Ethical approval

Ethical approval for this follow‐up assessment was given by the Medical Ethics Committee of the Academic Medical Center in Leiden (P1a. G18.121) (approval date cryopreservation 24 May 2006, additional approval follow‐up study 3 December 2018).

## 3 RESULTS

### 3.1 Subject characteristics and indications

**Figure 1:**
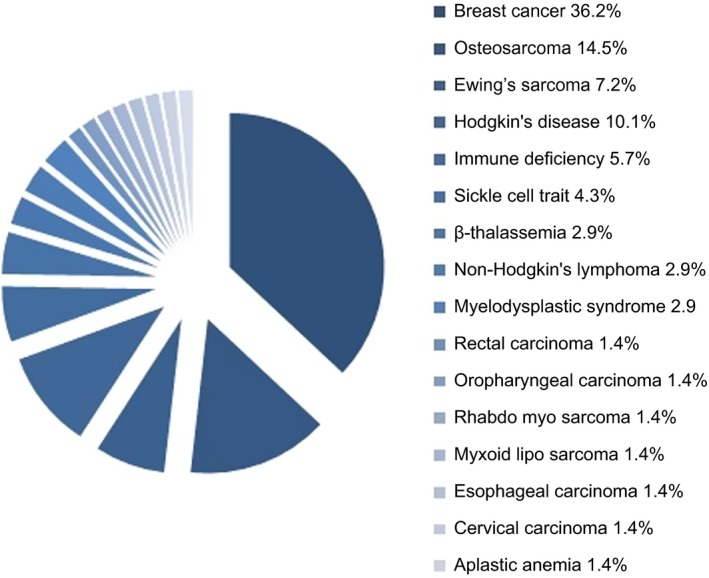
Indications for ovarian tissue cryopreservation [Color figure can be viewed at http://wileyonlinelibrary.com]

Between July 2002 and December 2015, 19 young women (<18 years old, two patients were premenarchal) and 50 adult women (≥18 years old) underwent OTC before gonadotoxic treatment. Patient characteristics are shown in Table 1. The mean age was 24.0 years (range 10.2‐35.7 years). The indications for ovarian cryopreservation varied and included malignant (81.1%) and benign (18.9%) conditions (Figure 1).

**Table 1:** Patient characteristics

All patients (n** = **69)	Number of patients (%)	Mean age (range min‐max) (y)
Indication		
Malignant	56 (81.2)	25.55 (13.8‐35.7)
Breast cancer	25 (36.2)	31.24 (21.2‐35.3)
Other malignant disease	31 (44.9)	20.96 (13.8‐35.7)
Benign	13 (18.9)	16.23 (10.2‐28.8)
Total	69 (100)	23.84 (10.2‐35.7)
Hormonal function		
Premenarchal	2 (2.9)	13.0 (11.5‐15.5)
Fertile	67 (97.1)	25.91 (10.2‐35.7)
Total	69 (100)	23.84 (10.2‐35.7)

In 2 women, OTC was performed despite a moderate to high risk of ovarian metastases: 1 had rectal cancer, and 1 was diagnosed with abdominal disease of the esophageal cancer during the laparoscopy to harvest ovarian tissue. Six women had already been treated with low‐risk gonadotoxic treatment, but these women were included because of normal ovarian function and the need for additional gonadotoxic treatment. In total, 3 minor complications occurred (4.3%): 1 woman (1.4%) had an injury to the bladder due to the laparoscopy that was diagnosed after surgery. An indwelling catheter was inserted for 14 days, which resulted in complete recovery. One laparoscopic procedure (1.4%) was converted to a mini‐laparotomy because of instrumental defects. In 1 woman (1.4%), the insertion of the uterus mobilizer resulted in a laceration of the vagina that had to be sutured. All 3 woman recovered completely.

### 3.2 Gonadotoxic treatment

Follow up to determine survival, course of the disease, ovarian function, and fertility over time was obtained until October 2018. Sixty‐seven women have completed the gonadotoxic treatment, one woman is still under treatment and in 1 woman gonadotoxic treatment for breast cancer was scheduled but she rejected this treatment after OTC and hormonal treatment were administered after lumpectomy.

**Figure 2:**
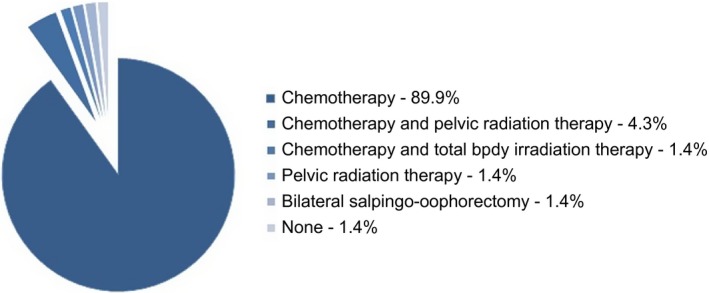
Primary treatment [Color figure can be viewed at http://wileyonlinelibrary.com]

The different regimens of gonadotoxic treatment are shown in Figure 2. After completion of gonadotoxic treatment, four of the 69 women (5.8%) underwent additional oophorectomy of the remaining ovary because of being *BRCA* mutation carriers (n = 3) or because of therapy‐resistant vaginal bleeding (n = 1).

### 3.3 Follow up

Follow‐up evaluation was performed until October 2018. The follow‐up time ranged from 4 to 183 months (mean 77.4 months). At time of follow up, 12 women had died (17.4%), so 57 (82.6%) were approached to answer questionnaires of whom 6 were lost to follow up (8.7%).

### 3.4 Survival

**Figure 3:**
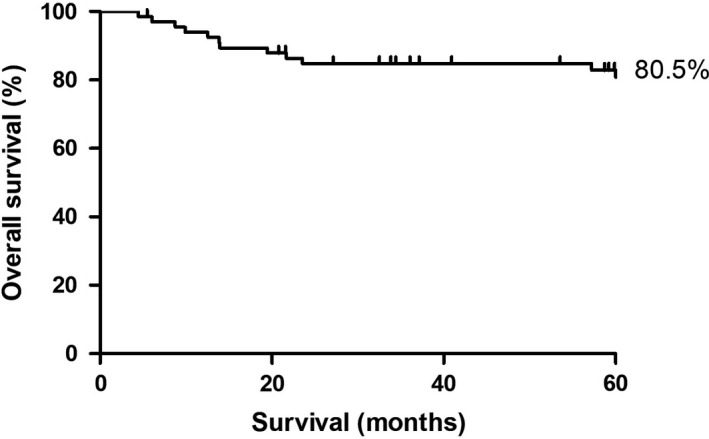
Five‐year survival analysis

The cumulative 5‐year survival rate of women who underwent OTC in our hospital is 80.5% (Figure 3). In total, 12 women died because of recurrence of disease, therapy resistance or after complications of hematopoietic stem cell transplantation.

### 3.5 Ovarian function

**Figure 4:**
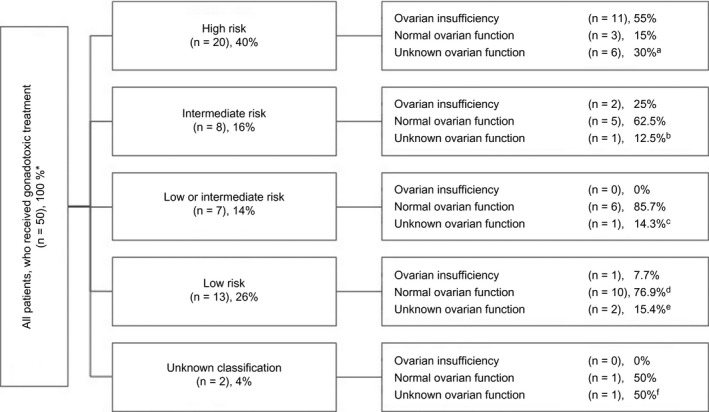
Ovarian function in relation to gonadotoxic treatment. ^*^All patients, diseased (n = 12) and lost to follow up (n = 5, excl. Bilateral salpingo‐oophorectomy (n = 1) and non‐gonadotoxic treatment (n = 1). ^a^Three women using contraception, 1 is still receiving treatment; ^b^1 woman uses contraception; ^c^1 woman uses contraception; ^d^4 women had normal ovarian function and underwent oophorectomy. ^e^1 woman uses contraception; ^f^1 woman is still receiving treatment

Of the 51 women, 20 (39.2%) developed POI: five of them because of bilateral salpingo‐oophorectomy after OTC. In the women who underwent bilateral salpingo‐oophorectomy after OTC, the removed ovary had normal function. Twenty‐three women (45.1%) have normal ovarian function. In our cohort, the percentage of women who developed POI due to gonadotoxic treatment was 29.4% (15/51). In women with breast cancer, this was 23.8% (5/21) and in women with osteosarcoma (n = 5) none suffered from POI as a result of gonadotoxic treatment. The ovarian function of the remaining eight (11.8%) women is unknown, as one of them is still premenarchal, six of them use hormonal contraception, and one is under treatment (Table 2). In Figure 4, the ovarian function in relation to different gonadotoxic regimens is displayed.

**Table 2:** Ovarian function after ovarian tissue cryopreservation and treatment

Ovarian function	All patients (n** = **51^a^) n – (%)	Breast cancer (n** = **21) n – (%)	Osteosarcoma (n** = **6) n – (%)
Premature ovarian insufficiency	20 – (39.2%)^b^	9 – (42.6%)^c^	0 (0%)
>12 mo amenorrhea and/or FSH > 40 IU/L	15	5	0
BSO/additional oophorectomy	4	3	0
Additional oophorectomy + hysterectomy	1	1	0
Normal ovarian function	23 – (45.1%)	11 – (52.4%)	5 – (83.3%)
Pregnant without transplantation	14 – (27.4%)	9 – (42.9%)	2 – (33%)
Unknown	8 – (11.8%)	1 – (4.8%)	1 – (16.7%)
Contraception (hormonal)	6	—	1
<1 y treatment	1	1	
Premenarchal	1	—	
Total	51 – (100%)	21 – (100%)	6 – (100%)

Note

One patient who is not included in the table, delivered a healthy baby and died afterward due to disease recurrence.

Abbreviations: BSO, bilateral salpingo‐oophorectomy; FSH, follicle‐stimulating hormone.

a Excludes 12 diseased, 6 lost to follow up.

b Corrected for BSO and oophorectomy of the remaining ovary = 15 (29.4%).

c Corrected for oophorectomy of the remaining ovary = 4 (23.8%).

Among all the women, 6 had already received low‐risk chemotherapy before OTC and were scheduled to receive hematopoietic stem cell transplantation treatment. Of the six women who underwent OTC despite earlier gonadotoxic treatment, two died (1 who had breast cancer and one with Hodgkin’s disease). Ovarian function of the other four was as follows: three women had POI (one with breast cancer who received two cycles of five fluorouracil; epirubicin; cyclophosphamide (FEC), 1 with Hodgkin’s disease who received adriamycine, bleomycine, vinblastine en dacarbazine (ABVD), and one with Hodgkin’s disease who received six cycles of epirubicine, bleomycine, vinblastine, prednison), and in one woman ovarian function was unknown (she had Hodgkin’s disease and received 8 cycles of ABVD).

Fifteen women (29.4%) conceived spontaneously after OTC without autotransplantation, which resulted in 25 deliveries. One woman died due to recurrence of the myxoid liposarcoma after she had delivered a healthy baby. Of these 15 women, nine had breast cancer (which accounts for 42.9% of the breast cancer patients) (Table 2).

### 3.6 Fertility after ovarian autotransplantation

In our follow‐up cohort, six women underwent 8 autotransplantations of cryopreserved ovarian tissue (Table 3). Additionally, one ovarian transplantation was performed in a woman in whom ovarian cryopreservation before gonadotoxic treatment for breast cancer had been carried out at another hospital. She was not included in our follow‐up cohort. The indications for OTC were breast cancer (n = 3), Hodgkin’s lymphoma (n = 2), non‐Hodgkin’s lymphoma (n = 1), and Ewing’s sarcoma (n = 1). Mean age at time of transplantation was 33.6 years (range 26.4‐40.1 years). Ovarian slices were placed back at orthotopic sites and once at a peritoneal window. The number of transplanted ovarian slices ranged from four to 12 (mean 8).

**Table 3:** Patient characteristics, ovarian function and fertility outcomes after cryopreserved ovarian tissue autotransplantation

	Patient 1^a^	Patient 2	Patient 3	Patient 4^a^	Patient 5	Patient 6	Patient 7^b^
Disease	Breast cancer	Breast cancer	Hodgkin lymphoma	Non‐Hodgkin lymphoma	Hodgkin lymphoma	Ewing’s sarcoma	Breast cancer
Age at OTC (y)	26	32	29	32	22	20	28
Age at transplantation (y)	31 33	35	39	39 40	26	26	38
Transplantation sites	Ovary Ovary	Ovary and peritoneal	Ovary	Ovary Ovary	Ovary	Ovary	Ovary
Transplanted ovarian slices (n)	11 11	4	7	8 8	12	6	5
Time to restoration of menstrual cycle (mo)	3.7	6.0	Not restored	3.4 3.0	2.1	2.4	3.0
Duration of ovarian function (mo)	Ovarian resection (*BRCA1*) (38.5)	Ongoing (45.0)	—	Ongoing (47.8)	Ongoing (57.3)	Ongoing (3.3)	Ongoing (15)
Pregnancy (n)	1	2	—	—	2	—	1
Fertility techniques	None	IVF None	—	—	IVF None	—	None
Time to first pregnancy (mo)	24	8	—	—	23	—	6
Pregnancy outcomes	1 live birth (followed by ovarian resection due to *BRCA1*)	2 live births	—	—	1 missed abortion, 2 live births	—	1 live birth

Abbreviation: IVF, in vitro fertilization; OTC, ovarian tissue cryopreservation.

a These women underwent 2 autotransplantation procedures.

b Patient recruitment and ovarian cryopreservation were performed in another hospital.

One woman did not benefit from the transplantation because ovarian function was not restored. In five women the transplants are still active at closure of follow up of this study, one woman underwent ovariectomy after delivery because of *BRCA1* mutation. A total of seven pregnancies occurred in four of these women, which resulted in 6 term deliveries and one miscarriage. Two pregnancies were accomplished after in vitro fertilization: in one woman one oocyte was retrieved in a spontaneous cycle, which was fertilized and resulted in one live birth. In the other woman, eight oocytes were retrieved in a stimulated cycle, of which one was fertilized. However this pregnancy resulted in a missed abortion.

## 4 DISCUSSION

We showed that ovarian cryopreservation with subsequent autotransplantation is a safe and successful technique with six live births in four out of seven patients (57%). Additionally, our previous data showed no ovarian metastases in ovarian tissue.^26^ The number of international reports and publications is increasing and OTC has been described in large series.^13^, ^14^ The usage rate of cryopreserved ovarian tissue transplantation (1.9%‐4%) and success rates with regard to live‐birth rate (31%‐37%) have been described.^12^, ^13^, ^14^, ^16^, ^27^, ^28^, ^29^ In this series of women who underwent OTC, the autotransplantation rate was 8.7% (6/69), the restoration rate of ovarian function was 86% (6/7 patients, one external patient included) and live‐birth rate after autotransplantation was 57% (4/7 patients).

One might argue that the usage rate of 8.7% might be different if patients were included according to the Edinburgh Criteria developed by Wallace et al.^23^ However, this hardly affected our results. The only difference with the Edinburgh Criteria compared with the inclusion criteria used in this study is the exclusion of patients >35 years. In our study, four out of all 69 women were aged 35 or 36. None of these four women developed POI or needed autotransplantation of the ovarian tissue. Hence, if we had followed the Edinburgh Criteria, the 45.2% with normal ovarian function would increase to 48.9% and the usage rate would increase from 8.7% to 10.8%.

Most women who underwent OTC had breast cancer (36%, n = 25). We used two main inclusion criteria: first a >50% risk of POI due to gonadotoxic treatment, second an estimated 5‐year survival rate of >50%. Given the 5‐year cumulative survival rate of our cohort (80.5%), the survival criteria were well used. However, estimation of survival is very difficult at the time of referral for discussions and decisions about fertility preservation.

In our study, 20 women (39.2%) developed POI after gonadotoxic treatment. We used the POI risk assessment initially published by Le Presti, Meirow and Di Cosino and updated by the American Society of Clinical Oncology in 2006 to guide whether OTC should be offered.^20^ However, other factors contributing to the risk of POI due to gonadotoxic treatment (eg age, anti‐Müllerian hormone and inhibin levels, and previous chemotherapy or irradiation of the pelvic area) are not taken into account in these assessments.^30^, ^31^, ^32^ Our results show that the risk of POI was overestimated at the time of OTC. The American Society of Clinical Oncology recommendation uses a 3‐tier system: high (>80%), intermediate (20%‐80%), and low (<20%) risk. In our cohort, the risks of POI after high‐, intermediate‐, and low‐risk regimens were respectively 55%, 25%, and 7.7% (Figure 4). Hence, some treatment regimens were inconclusive as to whether they should been assigned to low or intermediate risk and therefore a 4th group named low/intermediate was added, with none of the patients suffering from POI.

Additionally, despite an estimated risk of POI >50%, the women with breast cancer and those with osteosarcoma showed a low percentage of POI (23.8% and 0%, respectively), after gonadotoxic treatment. Andersen et al reported 60% of women with breast cancer had little evidence of ovarian damage.^15^ Also Petrek et al^33^ found that breast cancer patients <35 years had an approximately 85% recovery in monthly bleeding whereas women aged between 35 and 40 years recovery of monthly bleeding ranged from 45% to 61%. Data on female infertility following osteosarcoma therapy are limited, in 1 study, 6% of female patients treated with methotrexate, adriamycine and cisplatin plus Ifosfamide experienced early menopause.^34^ On the other hand, Larsen et al^35^ reported diminished ovarian reserve in young cancer survivors with spontaneous menstrual cycles. The risk of POI in these women was increased by a factor of four in teenagers and a factor of 27 in women between the ages of 21 and 25 years. Clearly treatment regimens of these patients should be classified as low or intermediate risk and perhaps OTC should not be offered. This is in parallel with the most recent recommendation of the International Society for Fertility Preservation Practice committee, which recommends OTC in women with breast cancer only when immediate gonadotoxic treatment is necessary.^36^ Whenever possible, according to this recommendation, these women should be counseled to cryopreserve embryos or oocytes. The ovarian stimulation needed for cryopreservation of oocytes or embryos must be counterbalanced in breast cancer patients with Letrozole to prevent high estrogen levels and the risk of cancer recurrence.^37^, ^38^, ^39^ When cryopreservation of embryos or oocytes is not possible, we advise discussion of OTC.

We have performed nine autotransplantations of cryopreserved ovarian tissue in seven women (6/69 patients [8.7%], and one external patient). Three women were breast cancer patients who had received gonadotoxic treatment with high risk of developing POI. Two of the seven patients had a second autotransplantation due to exhaustion of the first transplant. Because of the risk of exhaustion of the transplant, not all cryopreserved ovarian slices are transplanted in the first transplantation. In this way, it is possible to perform a second and, if necessary, a third transplantation. Worldwide, several second and/or third autotransplantations have been performed with successful restoration of ovarian function and good fertility outcomes.^5^ The first birth after autotransplantation of previously cryopreserved ovarian tissue in our center was in November 2015. Thereafter, six other pregnancies occurred which resulted in a total of five live births and one missed abortion. Pacheco and Oktay reported a meta‐analysis with success rates of 57.5% life‐birth rate, 37.7% ongoing pregnancy rate and an endocrine restoration rate of 63.9%.^40^ However, the meta‐analysis did not rule out underreporting of failed cases by others. Although our study describes small numbers of patients, OTC with subsequent transplantation is a successful technique with an ongoing pregnancy rate of 57%.

When looking at our results (and we have to realize that the usage rates at this moment might be an underestimation as OTC), not all women actively wish for a child and a large number of women in our cohort are still at risk of developing premature failure later in life: resumption of cyclic menses after high gonadotoxic treatment does not guarantee normal fertility.^41^ These women are future candidates for autotransplantation (resp. 8‐41 patients). In addition, the risk of POI needs to be elaborated. Our data may help women in the future and their physicians to decide about the options to preserve fertility in case of scheduled gonadotoxic treatment.

## 5 CONCLUSION

We show that autotransplantation of cryopreserved ovarian tissue is an effective method (restoration of ovarian function 86%) and has a live‐birth rate of 57% (4/7). However, given our low risk of POI after gonadotoxic treatment (29.4% normal ovarian function), the usage rate was only 8.7% (6/69). Especially in breast cancer, and perhaps in osteosarcoma, the risk of POI after gonadotoxic treatment was significantly lower than estimated. One can argue that fertility preservation is not necessary in these patients because of the low risk of POI and high chance of (spontaneous) pregnancy. Our data may help future patients and physicians in their discussions and decisions about the need and possibilities to preserve fertility. This will lead to an increase in the efficiency and applicability of care. Finally we would make a plea for an international collaboration to expedite the knowledge on need, safety, and effectiveness of OTC and autotransplantation.

## CONFLICT OF INTEREST

The authors have stated explicitly that there are no conflicts of interest in connection with this article.
